# Disclosure of Long-Term Complications in Informed Consent for Adolescent Idiopathic Scoliosis Undergoing Posterior Spinal Fusion Surgery: A Systematic Review of Online Resources

**DOI:** 10.3390/jcm15093210

**Published:** 2026-04-23

**Authors:** Carlos Barrios, Jesús Burgos, Eduardo Hevia, Vicente García, Hashem Altabbaa, Gonzalo Mariscal

**Affiliations:** 1 Institute for Research on Musculoskeletal Disorders, Valencia Catholic University, 46001 Valencia, Spain; carlos.barrios@ucv.es; 2 Vithas Internacional, 28043 Madrid, Spain; jburgosflores@hotmail.com; 3 Spine Unit, University of Navarra Clinic, 28027 Madrid, Spain; eduardoheviasierra@gmail.com; 4 Spine Surgery Section, Araba University Hospital, 01009 Vitoria, Spain; vigarto@hotmail.com; 5 Al Basheer Hospital, Amman 11118, Jordan; hashem.altabbaa.20@gmail.com

**Keywords:** adolescent idiopathic scoliosis, posterior spinal fusion, informed consent, long-term complications, patient education, systematic review

## Abstract

**Background**: Posterior spinal fusion (PSF) for adolescent idiopathic scoliosis (AIS) is a standard procedure with recognized long-term complications that may emerge years after surgery. Informed consent requires disclosure of material risks, but it is unclear whether these long-term sequelae are consistently communicated. This study systematically reviewed publicly available consent materials to assess disclosure of evidence-based long-term complications of PSF for AIS. **Methods**: Official websites of spine, orthopedic, and neurosurgical societies, along with major hospitals across North America, South America, Europe, and Australia, were searched for publicly available informed consent forms and patient information leaflets related to PSF for AIS. Documents were assessed for explicit mention of predefined long-term complications: chronic pain/health-related quality of life, pseudoarthrosis, adjacent segment degeneration, future surgery, pulmonary function impact, late infection, local tissue reaction to metal debris, and pregnancy-related issues. Disclosure frequencies were calculated. **Results**: Thirty-one documents from ten countries were included. Immediate perioperative risks were almost universally reported, whereas long-term complications were inconsistently disclosed. Reporting frequencies were: pseudoarthrosis, 80.6% (n = 25); future surgery, 67.7% (n = 21); adjacent segment degeneration, 51.6% (n = 16); chronic pain, 48.4% (n = 15); local tissue reaction to metal debris, 38.7% (n = 12); late infection, 25.8% (n = 8); pregnancy-related issues, 22.6% (n = 7); and pulmonary impact, 9.7% (n = 3). **Conclusions**: Publicly available consent materials for AIS surgery incompletely disclose long-term complications compared with the published evidence. However, written information sheets and consent forms represent only one component of the consent process. Consistently with the patient-centered standard articulated in Montgomery v Lanarkshire Health Board, informed consent should include discussion of material risks, benefits, reasonable alternative treatments including standard care, and the option of no treatment, with disclosure tailored to what matters to the patient and family. Updating written materials to better reflect lifelong risks may strengthen one important component of informed consent and shared decision-making for patients and families.

## 1. Introduction

Adolescent Idiopathic Scoliosis (AIS) is a three-dimensional spinal deformity that presents in otherwise healthy adolescents. For curves that demonstrate significant progression, posterior spinal fusion (PSF) with instrumentation is the established gold standard of surgical care [1]. The primary goals of this procedure are to halt curve progression, achieve coronal and sagittal plane correction, and provide a stable, solid arthrodesis. For the majority of these young patients, the spinal instrumentation is intended to be a lifelong implant, meaning they will live with a fused spine for many decades [2,3,4].

The ethical and legal cornerstone of any surgical intervention is the process of informed consent. Rooted in the principle of patient autonomy, Informed consent is not merely a signature on a form but an ongoing dialogue between the surgeon and the patient (or their legal guardians) [5,6,7]. This process should include adequate time for the patient and family to reflect on the information provided, the opportunity to seek a second opinion if desired before proceeding with treatment, confirmation of consent on the day of the procedure, and the patient’s freedom to withdraw consent or request additional information at any time. Consent may also be provided with specific patient preferences or caveats, reflecting individual values and priorities, and should be reconfirmed on the day of the procedure. This process is designed to ensure that the patient has a sufficient understanding of their diagnosis, the proposed treatment, its potential benefits, and, critically, its material risks and available alternatives [8]. This patient-centered approach is consistent with Montgomery v Lanarkshire Health Board (2015) UKSC 11, in which the UK Supreme Court held that clinicians must take reasonable care to ensure that patients are aware of material risks in the recommended treatment and of reasonable alternative or variant treatments. In this framework, a risk is material not only when a reasonable person in the patient’s position would likely attach significance to it, but also when the clinician is or should reasonably be aware that the particular patient would likely attach significance to it. Thus, informed consent requires attention not only to statistical risk disclosure, but also to what matters most to the patient and family in the context of decision-making. Such information should be communicated by the treating health professional, with written materials serving as supportive, not substitute tools within the consent process. However, the effectiveness of this process is often challenged. Studies have shown that patient comprehension of surgical risks can be remarkably low, with patients often recalling less than half of the information discussed [9,10]. This gap between the information provided and the information retained underscores the complexities of achieving truly informed consent [11,12].

While the immediate, perioperative risks of PSF—such as neurologic injury, infection, and blood loss—are standard components of the consent discussion, a growing body of evidence from long-term follow-up studies has illuminated a distinct profile of delayed-onset sequelae. As large cohorts of AIS patients now reach 20, 30, and 40 years post-surgery, research has consistently documented long-term challenges, including a higher prevalence of chronic back pain, accelerated adjacent segment degeneration, the need for late reoperation, and specific challenges related to pregnancy and childbirth [13,14,15]. These long-term outcomes constitute a critical part of the lifelong patient experience.

This raises a crucial question: is the consent process for AIS surgery adequately evolving to incorporate this wealth of long-term evidence? Given the documented challenges in patient comprehension and the expanding knowledge of delayed complications, it is unclear whether standard informed consent materials accurately reflect the full, decades-long trajectory of living with a fused spine. We therefore hypothesized that a gap exists between the long-term risks documented in the scientific literature and their disclosure in publicly available patient consent documents. The objective of this study was to systematically review publicly available written consent materials from professional spine societies and major institutions to quantify the disclosure rate of specific, evidence-based long-term complications of PSF for AIS. We recognize that these materials represent only one component of the broader informed consent process, which also includes verbal discussion, individualized counseling, and time for patient and family reflection.

## 2. Materials and Methods

This study was conducted as a systematic review of publicly available online patient resources. The objective was to identify and analyze informed consent documents, patient information sheets, and similar materials pertaining to PSF for AIS patients. The primary objective was to identify and analyze informed consent documents to assess the extent to which evidence-based, long-term complications of posterior spinal fusion (PSF) are disclosed to patients and their families.

### 2.1. Search Strategy

A systematic search was conducted to identify relevant documents. The search focused on the public-facing websites of national and international professional societies related to spine, orthopedic, and neurosurgery, as well as major hospitals and spine centers known for treating pediatric spinal deformities. The geographic scope was intentionally broad, targeting organizations in North and South America, Europe, and Australia to capture a diverse sample of international practices.

The search strategy involved two main approaches: (1) Direct Website Navigation: The official websites of prominent organizations (e.g., Scoliosis Research Society [SRS], North American Spine Society [NASS], British Association of Spine Surgeons [BASS], EUROSPINE) were manually explored. Website sections labeled “Patient Information,” “Public Resources,” “For Patients,” or “Consent Forms” were specifically examined. (2) Targeted Web Searches: A standardized search protocol using the Google search engine was employed with site-specific search operators to query each target domain. The following search string, translated and adapted as needed, was used: (site:URL) AND (“informed consent” OR “consent form” OR “risks of surgery”) AND (“scoliosis” OR “spinal fusion” OR “arthrodesis” OR PSF OR posterior spinal fusion).

Despite a broad search, publicly available consent forms could not be located for every targeted country, such as Portugal, due to limited online accessibility of such documents.

### 2.2. Eligibility Criteria

The PICO framework for this review was defined as follows:

Population: Adolescents with idiopathic scoliosis (and/or their legal guardians) for whom PSF is a potential treatment.

Intervention: Publicly available written informed consent documents (forms, templates, patient information leaflets) related to PSF or spine surgeries.

Comparator: A benchmark list of evidence-based long-term complications derived from the peer-reviewed literature, as detailed in Section 3. 

The Evidence Base: Long-Term complications of PSF for AIS.

Outcome: The documented disclosure (presence or absence) of specific long-term complications within the analyzed documents.

### 2.3. Inclusion and Exclusion Criteria

To be included in the systematic review, a document had to be an official informed consent template, a formal patient information sheet, or a detailed web-based resource that explicitly enumerated the risks associated with PSF for scoliosis. In practice, it is often the case that a general spinal arthrodesis consent form is used where scoliosis is a specified indication, rather than a form exclusively for PSF in AIS; such documents were included to ensure a comprehensive analysis. Eligible documents were required to be publicly accessible from a recognized professional society or hospital. Documents in English, Spanish, Portuguese, German, and French were considered for inclusion and translated into English for analysis.

Conversely, documents were systematically excluded if they pertained exclusively to non-idiopathic scoliosis (e.g., neuromuscular), non-fusion techniques (e.g., vertebral body tethering), or anterior-only surgical approaches. General website content such as blog posts, patient testimonials, marketing materials, and purely educational articles not structured as a formal risk disclosure were also excluded. Finally, any document requiring a membership login or payment for access was excluded.

### 2.4. Data Extraction and Analysis

Two reviewers independently screened all retrieved documents for eligibility based on the predefined criteria. Any discrepancies regarding document inclusion were resolved by discussion and consensus. A comprehensive file containing all included documents was compiled.

For each document that met the inclusion criteria, the following information was extracted into a standardized spreadsheet: (1) the source institution or society, (2) the country of origin, and (3) the document type (classified as “Informed Consent Form” or “Patient Information Leaflet”). The primary outcome was the explicit mention of specific complications. A binary (yes/no) data extraction was performed for a pre-defined list of both perioperative and long-term risks.

The list of long-term complications was derived from the evidence base established in the literature review (Section 3) and included: (1) pseudoarthrosis (failure of fusion), (2) need for future/additional surgery, (3) adjacent segment degeneration (ASD) (or equivalent lay terms such as “arthritis above/below the fusion” or “wear and tear on other discs”), (4) chronic pain and health-related quality of life, (5) pregnancy & childbirth-related issues, (6) late-onset infection (occurring months or years after surgery), (7) long-term impact on pulmonary function, and (8) local tissue reaction to metal debris (including related terms such as implant corrosion, metal debris, metal reaction, adverse local tissue reaction, or pseudotumor).

The frequency of disclosure for each complication was determined by tallying the number of documents in which it was mentioned. The data were synthesized descriptively and presented in a summary table to illustrate the disclosure patterns across the international sample.

## 3. The Evidence Base: Long-Term Complications of PSF for AIS

While PSF for AIS is highly effective in controlling spinal deformity, the decision to undergo this procedure involves accepting a lifelong alteration of spinal biomechanics [16]. As cohorts of patients treated with modern segmental instrumentation now reach follow-up periods of 10, 20, and even 30 years, a comprehensive profile of the long-term complications has become clearer. These outcomes extend beyond the immediate surgical period and encompass chronic pain syndromes, progressive radiographic changes, physiological adaptations, and a persistent risk of reoperation that collectively define the long-term patient experience [14].

### 3.1. Chronic Pain and Health-Related Quality of Life

A consistent theme across the long-term literature is the prevalence of chronic back pain and a measurable, albeit often non-disabling, reduction in quality of life compared to healthy peers. At a mean 23-year follow-up, Danielsson et al. [17] found that surgically treated patients reported significantly more lumbar pain (65%) than age- and sex-matched controls (47%), although the average pain intensity was mild. A meta-analysis by Lykissas et al. [18] noted that despite good surgical correction, long-term functional outcomes could be compromised. This is reflected in studies using validated patient-reported outcome measures (PROMs). At a 5-year follow-up, Helenius et al. [19] observed that while surgically treated patients had better pain and self-image scores than untreated AIS patients, their function scores remained significantly lower than those of healthy controls. Similarly, a study of middle-aged patients with over 21 years of follow-up found significantly lower scores for function and self-image compared to controls, even though pain and mental health scores were comparable [20]. A recent meta-analysis by Hevia et al. [21] confirmed these findings, showing that at ≥10 years post-surgery, AIS patients had significantly lower quality of life and higher disability than healthy peers across multiple domains, including pain and function, despite improvements in self-image. This suggests that while surgery prevents the worst outcomes of progressive deformity, it does not fully restore a normative state of physical function in the long term.

The cosmetic correction of the deformity is a major driver for surgery, and its impact on a patient’s self-perception is a key outcome. Long-term studies show mixed results. Akazawa et al. [20] found that middle-aged patients who had surgery decades prior still had significantly lower self-image scores on the SRS-22 compared to healthy controls. However, a study from Abdelaziz et al. [22] showed a dramatic improvement in the self-image domain of the SRS-22 after surgery, which was the domain with the highest increase. This suggests that while surgery significantly improves self-image from the preoperative state, it may not completely normalize it to the level of individuals who have never had a spinal deformity. The persistence of a rib hump or trunk asymmetry, even after surgery, can continue to affect body image long into adulthood [23]. Therefore, this impairment in quality of life may already be present before surgery and may not improve afterward; in some cases, it may even worsen.

### 3.2. Pseudoarthrosis

Pseudoarthrosis, also known as non-union, represents a critical long-term complication of PSF, characterized by the failure of the surgically treated spinal segments to form a solid, stable bone mass [24,25]. This failure creates a site of abnormal motion, which can lead to the persistence or recurrence of pain, loss of the initial surgical correction, and mechanical failure of the spinal instrumentation, such as rod or screw breakage [26,27]. Consequently, pseudoarthrosis is one of the primary indications for late reoperation following AIS surgery [28,29,30]. Furthermore, the micromotion at a pseudoarthrotic site is a significant contributing factor to instrumentation wear, corrosion, and the subsequent development of local tissue reaction to metal debris, creating a vicious cycle where mechanical failure and biological reactions amplify each other [31].

### 3.3. Adjacent Segment Degeneration

Perhaps the most studied long-term biomechanical consequence of spinal fusion is the accelerated degeneration of the adjacent mobile segments. The rigid fusion acts as a long lever arm, concentrating forces on the first unfused discs, which can hasten the natural aging process [16]. Long-term MRI studies have provided clear, objective evidence of this phenomenon. A landmark study by Green et al. [32] with a mean 11.8-year follow-up found an 85% incidence of new degenerative changes on MRI, noting that the L5-S1 disc was the most pathologically affected level, regardless of the lowest instrumented vertebra (LIV).

This finding has been consistently replicated. Nohara et al. [33] reported disc degeneration in 48% of patients at a minimum 10-year follow-up, with the incidence increasing significantly as the LIV was placed more caudally (from 34% at L1 to 68% at L3). A recent meta-analysis confirmed that the incidence of adjacent disc degeneration increases significantly with time, reaching 32% at an average of 13.8 years after surgery, with fusion below L3 being a significant risk factor [34].

In an MRI study at 11.4 years, found significantly higher rates of both disc and facet joint degeneration in AIS patients compared to controls, especially at the L1-L2 and L2-L3 levels just below the fusion [35]. Expanding on this study with a cohort with a mean 36-year follow-up, found that Modic changes (vertebral endplate signal abnormalities strongly associated with back pain) were present in 57.7% of AIS patients versus only 13.8% of healthy controls [20]. The presence of these changes correlated with a larger unfused lumbar curve and significantly worse ODI scores.

### 3.4. Long-Term Pulmonary Function

Surgical correction of thoracic AIS is often motivated by the goal of preserving pulmonary function. However, long-term studies indicate that for patients with moderate curves, surgery does not typically lead to an improvement in lung function relative to predicted normal values. A systematic review by Kato et al. [36] found no significant improvement in percent-predicted FVC (%FVC) or FEV1 (%FEV1) after posterior fusion at a minimum 2-year follow-up. This is borne out over longer time horizons. Min et al. [37] found that %FVC was unchanged from preoperative values at 10-year follow-up. Similarly, Byun et al. [38] reported no significant difference in percent-predicted FVC and FEV1 between preoperative measurements and those taken at a mean 15-year follow-up. Their analysis did suggest, however, that patients who presented with severe pulmonary impairment preoperatively were more likely to see a significant improvement, indicating a restorative rather than enhancing effect of surgery. A rigorous 2025 meta-analysis by Burgos et al. [39] confirmed this, demonstrating that PSF does not enhance respiratory function in AIS patients in either the short- or long-term. Therefore, this impairment in pulmonary function may already be present before surgery and may not improve afterward.

### 3.5. Need for Future/Additional Surgery

The concept of scoliosis surgery as a single, definitive “one and done” procedure is challenged by long-term reoperation data. A large national database analysis by Gouzoulis et al. [27] identified a 9.6% reoperation rate within 10 years of the index PSF. While nearly half of these revisions occurred in the immediate postoperative period, a significant number (22.8%) took place between 2 and 10 years later. Infection was a primary driver, accounting for nearly 30% of all reoperations.

These rates are consistent with prospective multicenter data. Hariharan et al. [40], reporting on a cohort with a minimum 10-year follow-up, found an overall major complication rate of 9.9% and a reoperation rate of 6.0%. Critically, they noted that complications such as deep surgical site infection and adding-on presented throughout the 10-year surveillance period, highlighting the necessity of long-term follow-up. Bartley et al. [41] previously reported on the same cohort at an earlier time point, finding a 4.1% rate of delayed major complications occurring 2 or more years after surgery, with wound problems (1.9%) and instrumentation-related issues (0.8%) being the most common causes. The reasons for late revision surgery are diverse and include pseudarthrosis (failure of fusion), painful or broken instrumentation, and progressive deformity, including phenomena like “adding-on” where the curve progresses below the fused levels [18,25].

### 3.6. Late-Onset Infection

Late-onset infection is a serious, delayed complication that can manifest months to years after the initial surgery, long after the primary surgical wound has healed [42]. Unlike acute postoperative infections, these are often caused by less virulent organisms, such as Propionibacterium acnes, which form a protective biofilm on the spinal implants, making them resistant to conventional antibiotic therapy [43,44]. Clinically, it can present insidiously with symptoms like persistent pain, drainage from a sinus tract, or signs of implant loosening, often mimicking aseptic hardware failure or pseudoarthrosis [45]. Late-onset infection is a leading cause for reoperation. A large database analysis found that infection was the primary driver for nearly 30% of all reoperations within 10 years of the index surgery [27]. This is consistent with prospective data showing that deep surgical site infections can present throughout a 10-year surveillance period [40]. Management often requires aggressive surgical intervention, including thorough debridement and, in many cases, complete removal of the instrumentation to eradicate the biofilm, which may then necessitate a complex, staged revision surgery [28]. This underscores that the risk of infection does not end after the initial surgical wound has healed but remains a lifelong possibility for patients with spinal hardware.

### 3.7. Local Tissue Reaction to Metal Debris

Local tissue reaction to metal debris is a rare but serious long-term complication resulting from the corrosion and wear of spinal implants [46,47]. It involves the infiltration of periprosthetic soft tissues, and possibly adjacent bone marrow, by variable amounts of metal particulate debris derived from titanium and/or cobalt-chromium alloys, leading to an inflammatory response characterized predominantly by macrophage infiltration and fibroblast proliferation with collagen deposition, sometimes accompanied by lymphocytes, plasma cells, mast cells, and eosinophils. In some cases, this reaction may form a mass of variable size resembling the so-called ‘pseudotumors’ described in metal-on-metal hip arthroplasty [46,48,49]. This process is often triggered by tribocorrosion—a combination of mechanical wear (fretting) and chemical corrosion—at the modular junctions of the instrumentation, such as rod-screw interfaces [50,51]. Crucially, its development is frequently associated with underlying mechanical instability, such as micromovements or pseudoarthrosis, which accelerates the wear process [26,48].

Clinically, deposits of metal debris of variable extent can manifest months to many years after the initial surgery. They may present with symptoms ranging from chronic pain and implant loosening to severe, progressive neurologic deficits such as paraparesis due to neural compression by the mass formed by the periprosthetic tissue reaction [45,50,52]. Diagnosis is challenging preoperatively because CT and MRI are often limited by artifact, making heavy deposition of metal debris an intraoperative finding, identified by characteristic gray-black tissue staining [51]. However, studies have shown that elevated blood or serum metal ion levels may reflect systemic exposure to implant-derived metal debris, but they cannot distinguish between corrosion, abrasion, fretting, or other wear mechanisms and therefore should not be considered specific biomarkers of implant corrosion [53]. In addition to their local effects, elevated circulating metal ion levels may also raise concern regarding possible systemic exposure to implant-related debris, although the clinical significance of this finding in spinal instrumentation remains incompletely defined [53,54]. The presence of metallic wear debris is therefore not limited to a local tissue reaction but may also indicate a more complex underlying problem, such as construct instability or failure of fusion [47]. A recent systematic review and meta-analysis by Burgos et al. [54] found that blood metal ion levels, particularly titanium and chromium, were frequently higher after instrumented spinal fusion than in preoperative or control measurements, suggesting systemic dissemination of implant-derived metal products. However, threshold values for clinical relevance are not well established, and the potential systemic consequences of these elevated levels remain incompletely understood.

### 3.8. Pregnancy and Childbirth

For the predominantly female AIS population, questions regarding future childbearing are of paramount importance. Long-term studies are largely reassuring. Kino et al. [55], in a study with a mean 27.5-year follow-up, found that the marriage rates and number of children among surgically treated women were not significantly different from healthy controls or national averages. This indicates that the history of PSF does not appear to be a barrier to family formation. However, the same body of literature consistently reports a higher incidence of significant back pain during pregnancy and a markedly increased rate of cesarean sections. A meta-analysis by Hevia et al. [56] confirmed that surgically treated AIS patients have a higher risk of cesarean section and report more back pain during pregnancy compared to healthy controls, though not necessarily more than conservatively treated AIS patients. The direct reasons for the increased cesarean rate remain debated [16]. An additional theoretical concern is whether implant-derived metal ions may have implications for fetal exposure during pregnancy. Although direct evidence in women with spinal instrumentation is lacking, studies from the metal-on-metal hip arthroplasty literature suggest that cobalt and chromium ions can cross the placenta, but consistent adverse fetal effects have not been clearly demonstrated. Accordingly, any potential effect on fetal development after AIS spinal fusion remains uncertain and should be interpreted cautiously [57].

## 4. Results

### 4.1. Search Results and Document Characteristics

**Figure 1:**
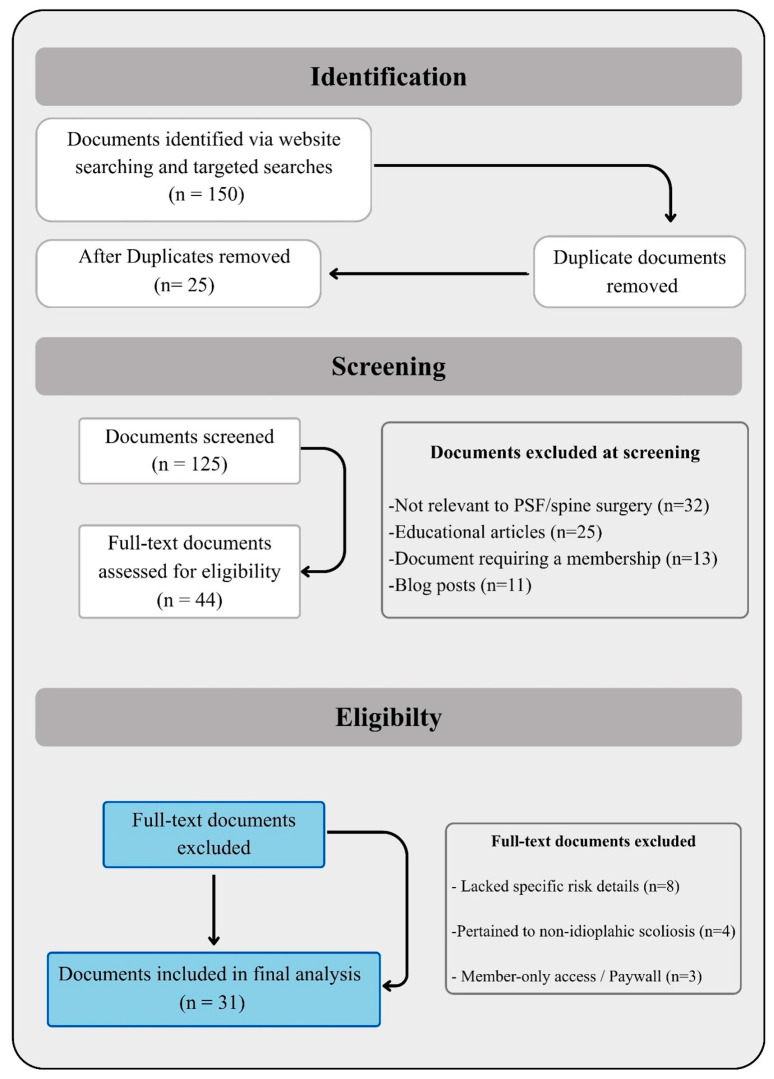
PRISMA flow diagram of the search process.

The systematic search of publicly accessible online resources yielded a total of 31 unique documents that met the full inclusion criteria. The PRISMA flow diagram is presented in Figure 1. These materials represent a diverse international sample, originating from institutions and professional societies in North and South America, Europe, and Australia. The collection includes 19 formal informed consent forms intended for patient signature and 12 detailed patient information leaflets or brochures.

Sources ranged from national professional bodies, such as the British Association of Spine Surgeons (BASS) and the Spanish Society of Orthopedic Surgery and Traumatology (SECOT), to large academic medical centers and regional health systems, including the Hospital for Special Surgery (USA), Sheffield Children’s NHS Foundation Trust (UK), and the Queensland Government (Australia). Documents published in Spanish, Brazilian Portuguese, German, Italian, and French were translated into English for a unified analysis.

### 4.2. Disclosure of Surgical Risks in Informed Consent Documents

The most commonly mentioned long-term issue was the potential for pseudoarthrosis (failure of fusion), which was noted in 80.6% (n = 25) of the documents. This was often linked to the need for future or additional surgery, a more general risk mentioned in 67.7% (n = 21) of the documents. Adjacent segment degeneration, often described in lay terms such as ‘deterioration of other discs’ or ‘wear and tear,’ was present in 51.6% (n = 16) of the documents. However, other critical long-term outcomes were mentioned far less frequently. Chronic back pain as a distinct entity persisting long after the initial recovery period was disclosed in 48.4% (n = 15) of the documents. Local tissue reaction to metal debris, implant corrosion, or equivalent related terminology was mentioned in 38.7% (n = 12) of the documents. Issues related to future pregnancy and childbirth were mentioned in 22.6% (n = 7) of the documents. The potential for long-term negative effects on pulmonary function was nearly absent, appearing in only 9.7% (n = 3) of the documents. Late-onset infection, occurring months or years after surgery, was specified in 25.8% (n = 8) of the documents. Table 1 provides a detailed breakdown of the findings for each institution and each of the targeted long-term complications.

The most commonly mentioned long-term issue was the potential for pseudoarthrosis (failure of fusion), which was noted in 25 of the 31 documents (80.6%). This was often linked to the need for future or additional surgery, a more general risk mentioned in 21 documents (67.7%). ASD, often described in lay terms such as “deterioration of other discs” or “wear and tear,” was present in 16 documents (51.6%). However, other critical long-term outcomes were mentioned far less frequently. Chronic back pain as a distinct entity persisting long after the initial recovery period was disclosed in only 15 of the 31 documents (48.4%). Local tissue reaction to metal debris or implant corrosion was mentioned in 12 documents (38.7%). Issues related to future pregnancy and childbirth were mentioned in just 7 documents (22.6%). The potential for long-term negative effects on pulmonary function was nearly absent, appearing in only 3 documents (9.7%). Late-onset infection, occurring months or years after surgery, was specified in 8 documents (25.8%). Table 1 provides a detailed breakdown of the findings for each institution and each of the targeted long-term complications.

## 5. Discussion

This systematic review of 31 publicly available informed consent documents from a diverse range of international institutions reveals a significant and consistent disparity: while the immediate, perioperative risks of PSF for AIS are almost universally disclosed, the well-documented, long-term sequelae are frequently underrepresented or entirely omitted. Our analysis demonstrates that although catastrophic acute risks like neurologic injury and infection are thoroughly addressed, chronic conditions such as adjacent segment degeneration, long-term pain, and challenges related to future pregnancies are mentioned in less than half of the materials provided to patients and families. This “information gap” suggests that the current standard of written informed consent may not adequately prepare patients for the lifelong implications of living with a fused spine. Importantly, our findings reflect only one part of the informed consent process—namely, what is disclosed in publicly available written materials—and do not directly measure the broader process of consent, including ongoing verbal discussions; individualized counseling; the opportunity for patients and families to ask questions; patient and family understanding; whether adequate time was provided for reflection before decision-making; the opportunity to seek a second opinion; continued discussion of benefits, risks, alternative treatments, and the option of no treatment; a detailed, patient-specific discussion of the proposed procedure; whether consent was given with specific patient preferences or caveats, whether consent was reconfirmed on the day of surgery, or whether additional information was provided over time in response to patient priorities, patient questions, evolving evidence, or changes in available alternatives; and the patient’s freedom to withdraw consent or request additional information at any time. Accordingly, the aim is not merely to provide more information, but to provide the information that is material for that specific patient: what outcomes, burdens, trade-offs, and future implications matter most to them.

### 5.1. The Disconnect Between Evidence and Practice in Informed Consent

Several practical strategies could improve the consistency and patient-centeredness of written consent materials for AIS surgery [5,8]. First, professional societies could develop standardized, evidence-based consent templates that include a core set of long-term sequelae (chronic pain, ASD, late infection, possible future surgery, pregnancy-related issues, and implant wear/local tissue reaction to metal debris), alongside common perioperative risks [5,8]. Second, institutions could supplement written forms with digital resources to ensure long-term risks reflect the evolving evidence base [11,12]. Finally, structured shared decision-making tools (brief question prompts for families and standardized counseling checklists) could support more consistent disclosure while allowing individualized discussion based on curve type, fusion levels, and patient priorities [5,8].

The core of informed consent is to support autonomous, patient-centered decision-making. This principle was articulated clearly in Montgomery v Lanarkshire Health Board (2015) UKSC 11, where the UK Supreme Court held that clinicians are under a duty to take reasonable care to ensure that patients are aware of any material risks involved in a recommended treatment, as well as any reasonable alternative or variant treatments. In that judgment, materiality depends not only on what a reasonable person in the patient’s position would want to know, but also on what the clinician knows—or should reasonably know—matters to the particular patient. Applied to AIS surgery, this means that consent should not be limited to a standard list of perioperative complications. Rather, it should include discussion of the expected benefits of surgery, short- and long-term risks, reasonable alternatives including standard non-operative care when applicable, and the option of no treatment/status quo, all framed in light of the adolescent patient’s and family’s priorities, concerns, and values.

Our analysis found that ASD was mentioned in only 16 of 31 documents. This stands in stark contrast to long-term MRI studies showing radiographic evidence of accelerated degeneration in up to 85% of patients at 10–20 years post-surgery [32,35]. Similarly, chronic back pain and its impact on quality of life, a theme prevalent across numerous long-term follow-up studies [17,19], was disclosed in only 15 of the documents. The nearly complete absence of information regarding long-term pulmonary function (mentioned in only 3 documents) and the low disclosure rate for pregnancy-related issues (mentioned in 7 documents) further highlight this disconnect.

This discrepancy may stem from several factors. Historically, the consent process has prioritized the immediate, high-impact risks of the surgical event itself, such as paralysis or death [8]. Furthermore, there may be a perception that discussing potential problems decades in the future could cause undue anxiety or information overload for an adolescent patient and their family [10]. However, the literature on patient comprehension suggests that the issue is not necessarily the volume of information but its clarity, relevance, and the method of delivery [12]. The lack of standardized, evidence-based consent templates from leading professional societies may also contribute to the variability and omissions observed in our study.

### 5.2. Implications for Patient Care and Shared Decision-Making

The findings of this review have significant implications for the modern practice of patient-centered care and shared decision-making. For an adolescent patient, the decision to undergo PSF is a decision with lifelong consequences. A truly shared decision-making process must extend beyond the immediate surgical risks and encompass a realistic discussion about what life may look like at age 40 or 50 with a fused spine.

Providing this long-term context is not about deterring patients from a necessary and beneficial surgery. Rather, it is about setting realistic lifelong expectations. A patient who understands that a higher-than-average likelihood of developing back pain or spinal arthritis is a known trade-off for a corrected spine is better prepared to manage those symptoms should they arise. This knowledge can transform future challenges from unexpected ‘complications’ into manageable, anticipated sequelae of a past intervention, which may improve long-term patient satisfaction. In addition, structured long-term support programs, including remote follow-up resources, educational materials, and online question-and-answer sessions, may help patients and families better understand and manage delayed complications over time.

Furthermore, studies have shown that patient comprehension of the consent process is often poor, with recall of discussed risks dropping significantly over time [9,11]. Supplementing the verbal consultation with clear, comprehensive written materials that include long-term outcomes can serve as a durable reference for the patient and their family, reinforcing the verbal discussion and improving long-term understanding.

### 5.3. Limitations

This study has several limitations. First, our review was restricted to publicly available documents. We did not contact individual institutions to request access to non-public consent forms. This approach was intentional, as the objective of the study was to evaluate materials that are readily accessible to patients and families outside the clinical setting. While direct institutional contact might have increased the number of documents analyzed, it could also introduce selection bias related to variable response rates and differences in institutional willingness to share internal materials. The consent forms obtained are not exhaustive and represent only what is accessible through public-facing websites. Second, and most importantly, the written consent form is only one component of the informed consent process. It cannot capture whether clinicians explored what mattered most to the patient and family, nor whether the discussion adequately addressed material risks, expected benefits, reasonable alternatives including standard care, and the option of no treatment/status quo. Informed consent is a dynamic interaction that includes detailed discussion between the surgeon, the patient, and their family, as well as time for reflection and shared decision-making. Written materials, including consent forms and patient information sheets, represent only a partial record of this process. In particular, they do not capture the information delivered directly by health professionals, which remains the core of the consent process. They also do not capture whether patients and families were given adequate opportunity to ask questions and receive tailored answers during the consent discussion. Likewise, our methodology cannot assess whether adequate time was provided for patients and families to consider the information before consenting to surgery, whether patients were offered or sought a second opinion as part of the decision-making process, whether ongoing discussions addressed benefits, risks, alternative treatments, and the option of no treatment, whether a detailed, patient-specific discussion of the proposed procedure was undertaken, whether consent was provided with specific patient preferences or caveats, whether consent was reconfirmed on the day of surgery, or whether additional information was provided at different stages in response to what mattered most to the patient, the questions raised by the patient and family, advances in knowledge, or the availability of alternative treatments. The crucial verbal dialogue, where many nuances, individualized risk discussions, and patient-specific considerations are addressed, could not be captured by our methodology. Therefore, our findings may overestimate the magnitude of the observed ‘information gap’ if long-term risks are routinely discussed during clinical consultations despite not being explicitly documented in written materials. However, written documents remain an important reference for patients and serve as the formal record of risk disclosure. Future research should therefore evaluate the entire consent process for AIS surgery, including surgeon-patient-family communication, decisional understanding, and the role of written materials within shared decision-making. In addition, we did not perform a formal quality assessment of the documents included (e.g., completeness, clarity, readability, or usability). This is because there is no validated, widely accepted tool specifically designed to assess the quality of informed consent documents for PSF in AIS across different institutions and languages, and applying non-specific instruments could introduce additional bias. In addition, our results are primarily descriptive. This review identifies which evidence-based long-term sequelae are underrepresented in publicly available written materials, but it does not provide a robust analysis of the underlying reasons for variability between regions or institutions. Likewise, while we propose general strategies to strengthen written consent resources, we did not test the feasibility, implementation barriers, or real-world effectiveness of specific remediation approaches. Future work should therefore explore the determinants of disclosure variability and evaluate targeted interventions to improve the consistency and patient-centeredness of written consent materials. Finally, our search was limited to documents in several major Western languages, and our findings may not be generalizable to all global regions.

## 6. Conclusions

This systematic review reveals a significant deficit in the written communication of evidence-based, long-term complications of posterior spinal fusion for AIS. While immediate perioperative risks are thoroughly disclosed, the long-term sequelae—including chronic pain, adjacent segment degeneration, and pregnancy-related challenges—are inconsistently mentioned in publicly available informed consent documents. This information gap within publicly available written materials may weaken one important component of fully informed, patient-centered, shared decision-making, particularly when consent should be guided by material risk and by what matters to the patient and family. Because informed consent extends beyond written documents alone, future work should assess how these materials interact with verbal counseling and the broader consent process in clinical practice. We recommend that professional spine societies and major institutions lead an effort to update their patient education materials and consent templates to include a clear, accessible, and evidence-based discussion of the known long-term outcomes. By doing so, the surgical community can better prepare patients not just for the operation, but for a lifetime with a fused spine.

## Figures and Tables

**Table 1:** Analysis of Long-Term Complication Disclosure in Informed Consent Documents.

Institution/Society	Country	Pseudoarthrosis (Non-Union)	Need for Future Surgery	Adjacent Segment Degeneration	Chronic Pain (Long-Term)	Pregnancy-Related Issues	Late-Onset Infection	Pulmonary Function (Long-Term)	Local Tissue Reaction to Metal Debris (Corrosion)
**NORTH AMERICA**									
Hospital for Special Surgery (HSS)	USA	Yes	Yes	Yes	Yes	No	No	No	Yes
MarinHealth Medical Center	USA	Yes	Yes	Yes	Yes	Yes	Yes	Yes	Yes
Penn State Health Children’s Hospital	USA	Yes	Yes	Yes	No	No	Yes	No	Yes
CHOC Children’s Orthopaedic Institute	USA	No	Yes	No	No	No	Yes	No	Yes
Colorado Orthopedic Centers of Excellence	USA	Yes	Yes	Yes	Yes	No	Yes	No	No
**EUROPE**									
British Association of Spine Surgeons (BASS)	UK	No	Yes	No	No	No	No	No	No
British Spine Registry	UK	No	No	No	No	No	No	No	No
The Spine Surgery London	UK	Yes	Yes	No	Yes	No	No	No	Yes
Sheffield Children’s NHS Foundation Trust	UK	Yes	Yes	No	Yes	No	Yes	Yes	No
Cambridge University Hospitals (NHS)	UK	No	Yes	No	Yes	No	Yes	No	Yes
Royal Devon University Healthcare (NHS)	UK	Yes	Yes	No	No	Yes	No	No	No
Spine Center Bielefeld (Wirbelsäulenzentrum)	Germany	Yes	Yes	Yes	Yes	No	Yes	No	Yes
The Foundation for the Prevention of Medical Risk	France	Yes	Yes	No	No	No	No	No	Yes
Spanish Society of Orth. Surg. (SECOT/GEER)	Spain	Yes	Yes	Yes	Yes	No	No	No	Yes
Spanish Association First in Health	Spain	Yes	Yes	Yes	No	No	No	Yes	Yes
Andalusian Health Service	Spain	No	No	No	No	No	No	No	No
Piccole Figlie Hospital	Italy	Yes	Yes	No	Yes	No	No	No	Yes
**SOUTH AMERICA**									
Albert Einstein Israelite Hospital	Brazil	Yes	Yes	Yes	Yes	No	No	No	Yes
Dona Helena Clinical Center	Brazil	Yes	Yes	No	Yes	No	No	No	Yes
10th of July Hospital	Brazil	Yes	No	No	Yes	No	No	No	No
Our Lady of Sorrows Hospital	Brazil	Yes	No	Yes	Yes	No	No	No	Yes
Medical Palmas/Santa Thereza	Brazil	Yes	No	Yes	Yes	No	No	No	Yes
Vitória Apart Hospital	Brazil	Yes	Yes	No	No	No	No	No	Yes
Hospital Orizonti	Brazil	Yes	Yes	Yes	Yes	No	No	No	No
Santa Casa de Marília	Brazil	Yes	Yes	Yes	Yes	No	No	No	Yes
Santa Helena Hospital	Brazil	Yes	Yes	No	Yes	No	No	No	No
Argentine Assoc. of Ortho. & Traumatology (AAOT)	Argentina	Yes	No	No	No	No	No	No	No
Neuquén Provincial Hospital	Argentina	Yes	Yes	Yes	Yes	No	No	No	Yes
The Polyclinic of Wellness (Spanish Assoc.)	Uruguay	Yes	No	No	No	No	No	No	No
**AUSTRALIA**									
Queensland Government Health	Australia	Yes	Yes	Yes	Yes	No	No	No	No
**Total (n = 31)**		**25**	**21**	**16**	**15**	**7**	**8**	**3**	**12**

Abbreviations: PSF, posterior spinal fusion; AIS, adolescent idiopathic scoliosis.

## Data Availability

No new data were created or analyzed in this study. Data sharing is not applicable to this article.

## Abbreviations

The following abbreviations are used in this manuscript:

AIS	Adolescent Idiopathic Scoliosis
ASD	Adjacent Segment Degeneration
CT	Computed Tomography
FEV1	Forced Expiratory Volume In 1 Second
FVC	Forced Vital Capacity
LIV	Lowest Instrumented Vertebra
MRI	Magnetic Resonance Imaging
ODI	Oswestry Disability Index
PSF	Posterior Spinal Fusion
